# Estimating the Societal Benefits of THA After Accounting for Work Status and Productivity: A Markov Model Approach

**DOI:** 10.1007/s11999-016-5084-9

**Published:** 2016-10-03

**Authors:** Lane Koenig, Qian Zhang, Matthew S. Austin, Berna Demiralp, Thomas K. Fehring, Chaoling Feng, Richard C. Mather, Jennifer T. Nguyen, Asha Saavoss, Bryan D. Springer, Adolph J. Yates

**Affiliations:** 1 KNG Health Consulting, LLC, 15245 Shady Grove Road, Suite 365, Rockville, MD 20850 USA; 2 Maryland Health Care Commission, Baltimore, MD USA; 3 The Rothman Institute, Philadelphia, PA USA; 4 OrthoCarolina, Charlotte, NC USA; 5 Duke Orthopaedic Surgery, Durham, NC USA; 6 University of Pittsburgh Medical Center, Pittsburgh, PA USA

## Abstract

**Background:**

Demand for total hip arthroplasty (THA) is high and expected to continue to grow during the next decade. Although much of this growth includes working-aged patients, cost-effectiveness studies on THA have not fully incorporated the productivity effects from surgery.

**Questions/Purposes:**

We asked: (1) What is the expected effect of THA on patients’ employment and earnings? (2) How does accounting for these effects influence the cost-effectiveness of THA relative to nonsurgical treatment?

**Methods:**

Taking a societal perspective, we used a Markov model to assess the overall cost-effectiveness of THA compared with nonsurgical treatment. We estimated direct medical costs using Medicare claims data and indirect costs (employment status and worker earnings) using regression models and nonparametric simulations. For direct costs, we estimated average spending 1 year before and after surgery. Spending estimates included physician and related services, hospital inpatient and outpatient care, and postacute care. For indirect costs, we estimated the relationship between functional status and productivity, using data from the National Health Interview Survey and regression analysis. Using regression coefficients and patient survey data, we ran a nonparametric simulation to estimate productivity (probability of working multiplied by earnings if working minus the value of missed work days) before and after THA. We used the Australian Orthopaedic Association National Joint Replacement Registry to obtain revision rates because it contained osteoarthritis-specific THA revision rates by age and gender, which were unavailable in other registry reports. Other model assumptions were extracted from a previously published cost-effectiveness analysis that included a comprehensive literature review. We incorporated all parameter estimates into Markov models to assess THA effects on quality-adjusted life years and lifetime costs. We conducted threshold and sensitivity analyses on direct costs, indirect costs, and revision rates to assess the robustness of our Markov model results.

**Results:**

Compared with nonsurgical treatments, THA increased average annual productivity of patients by USD 9503 (95% CI, USD 1446–USD 17,812). We found that THA increases average lifetime direct costs by USD 30,365, which were offset by USD 63,314 in lifetime savings from increased productivity. With net societal savings of USD 32,948 per patient, total lifetime societal savings were estimated at almost USD 10 billion from more than 300,000 THAs performed in the United States each year.

**Conclusions:**

Using a Markov model approach, we show that THA produces societal benefits that can offset the costs of THA. When comparing THA with other nonsurgical treatments, policymakers should consider the long-term benefits associated with increased productivity from surgery.

**Level of Evidence:**

Level III, economic and decision analysis.

**Electronic supplementary material:**

The online version of this article (doi:10.1007/s11999-016-5084-9) contains supplementary material, which is available to authorized users.

## Introduction

Total hip arthroplasty (THA) is a highly successful medical intervention, having favorable long-term outcomes in terms of improvement of physical functioning, survivorship, and self-reported quality of life [2, 6, 24]. The increased medical costs from surgery per quality-adjusted life year (QALY) gained compares favorably with those of other surgical procedures, such as lumbar discectomy, rotator cuff repair, and ACL repair [18, 21, 22, 25, 26, 34]. Chang et al. [9] showed that THA could be cost saving for some patients compared with nonoperative treatment for end-stage osteoarthritis (OA) after accounting for avoided costs for nursing homes and other long-term care.

Owing in part to the aging population and expanded indications, the number of THAs in the United States is expected to increase substantially. Across all patients, primary THA is projected to grow by 75% from 293,094 to 511,837 between 2010 and 2020 [20]. People younger than 65 years represent the fastest growing segment of patients having THAs, accounting for 47% of all THAs performed in 2012 compared with 34% in 1997 [15, 16]. One study projected that more than 50% of THAs will be performed in patients younger than 65 years by 2030 [19].

The current emphasis on reducing healthcare expenditures may cause payers to limit utilization of THA by implementing restrictive coverage or reimbursement policies. For example, in Oregon, a health plan for state workers has placed enhanced cost-sharing requirements on joint replacement, to reduce spending on the procedure. The consequences of such policies will depend on the societal benefits derived from THA. Because working-age patients represent a large number of all patients having THAs, potentially important indirect benefits from THA relate to work status, such as maintaining employment, reducing missed work days, and increasing workplace productivity [28]. However, we are unaware of any studies that account for work-related (indirect cost) benefits of surgery.

The goal of our study was to determine the net societal value of THA in the United States by estimating societal costs and benefits. We build on the approach used by Dall et al. [11] and Ruiz et al. [35], which links functional status and economic outcomes to changes in patient outcomes from surgery. The research addresses two questions: the effects of surgery on employment and earnings and how the inclusion of these factors influenced the measured cost-effectiveness of THA relative to nonsurgical treatment.

## Materials and Methods

**Fig. 1A–B:**
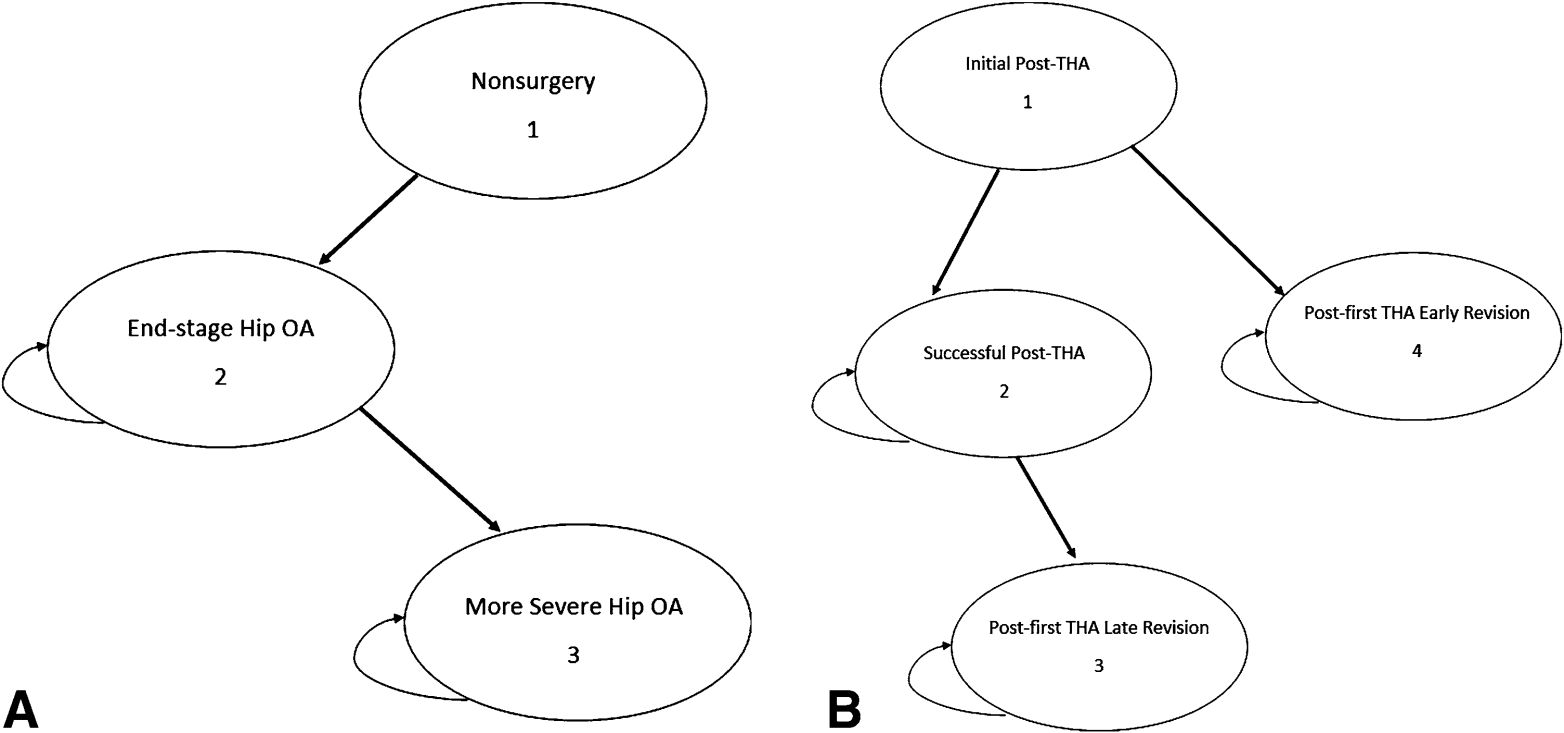
The Markov health state transition of both treatment arms is shown. **(A)** For nonoperative treatment, a patient enters a nonsurgery state and proceeds to end-stage hip OA, and then either remains there or transitions to a more severe state with greater functional impairment. **(B)** For surgical treatment (THA), a patient either dies or survives the primary THA. After initial surgery, the model assumes that individuals remain in a postprocedure state for 1 year (Initial Post-THA), which accounts for the costs and limitations of treatment and recovery and any complications. After surviving the initial (first year) post-THA state, patients either may enter the successful THA state or undergo a revision and thus enter the post-first THA early revision state. For patients entering successful THA state, they may remain in this health state or have late failure, undergo a revision, and thus enter the post-first THA late revision state. For patients entering the post-first THA early or late revision, they may remain in this health state or require a second revision, which was considered as a transitional health state (not seen in the Markov process).

We investigated the cost-effectiveness of THA compared with nonoperative treatment for patients with OA of the hip using a state-transition Markov decision model (Fig. 1). Benefits were estimated from the societal perspective for a patient cohort that had THAs. The cycle length was one year and the model cycled until patient death. The primary effectiveness outcome was expressed in QALYs. We used Medicare payments adjusted to an all-payer population to account for higher payment rates from private payers relative to Medicare. Costs and utilities were discounted at 3% per year. The model and analyses were performed using a decision analysis software package (TreeAge Pro; TreeAge Software Inc, Williamstown, MA, USA) (Appendix 1. Supplemental material is available with the online version of *CORR* ^®^.)

### Markov Model

Our decision tree consisted of two primary treatment arms: THA and nonoperative treatment. There were eight health states associated with the two treatment arms, including six Markov health states (Fig. 1), one transitional health state, and one absorbing state. In the Markov process for THA (Fig. 1B), post-first revision THA represents failure of the primary THA. The model differentiates between early and late failure of surgery and revisions attributable to infection and asepsis. Post-second THA revision is a transitional health state. A patient may transition to a second THA revision after entering the post-first THA revision state or stay in the post-first THA revision state. Death was an absorbing state that could occur at any point in the model based on the natural mortality rates from the US Census Bureau’s life expectancy tables [39]. If a patient transitions from a surgical state, the probability of death is a combination of the probability of natural mortality and surgical mortality, which varied by age and gender. Patients who enter the nonoperative treatment arm cannot cross over to the surgical arm. While this assumption might not reflect clinical reality, we imposed this requirement because we were interested in the value of access to THA.

### Model Assumptions

#### Indirect Costs

Our approach for estimating indirect costs was based on the methods used by Dall et al. [11]. We used National Health Interview Survey (NHIS) [7] data to generate regression coefficients that described the statistical relationship between physical functioning and economic outcomes. We then applied regression coefficients to patient-reported outcomes data to estimate the effects of surgery on the likelihood of employment, earnings, and missed work days.

The relationship between earnings and functional status was estimated using a two-stage Heckman selection model to correct for potential bias created because earnings are not observed for nonemployed individuals. We used a probit model to predict employment and a linear regression equation to predict earnings. The explanatory variables in both models included age, race, marital status, family size, education, and functional status indicators. Other sources of income and the presence of other working household members were included in the employment model. We used multiple imputation techniques to correct for the deflated standard errors that resulted from imputing personal income data in the NHIS [37].

Retrospective patient-reported outcomes data were obtained at two large physician group practices. Patients were asked to report on functional limitations using questions from the NHIS, which relate to ability to walk a quarter mile, climb 10 steps, sit for 2 hours, stand for 2 hours, stoop, carry a 10-pound bag, and push a large object. Respondents were asked to report functional ability at the time of the survey (followup) and to recall their function just before THA (baseline). We obtained 77 usable responses from patients (average age, 60 years; SD, 10.94 years) who generally received the survey 12 to 24 months after THA (Appendix 1. Supplemental material is available with the online version of *CORR* ^®^).

We used a simulation-based approach to estimate the change in productivity between baseline and followup. For each iteration, we sampled 77 patients (with replacements) from the patient-reported outcomes dataset. We then applied patient responses to the functional assessment questions to the Heckman model parameter estimates to calculate the probability of employment and earnings conditional on employment at baseline and followup for each individual in the sample. We ran 1000 iterations of the simulation. For each sample, we calculated the change in mean productivity (equal to expected earnings) between baseline and followup. We then calculated a mean change in productivity across all 1000 samples and the 95% CI using the distribution of mean change in productivity.

To generate results by age group and gender, we used the functional status of the original sample of 77 responses and predicted the probability of employment and earnings by altering respondents’ age and gender. We used this approach because employment and earnings are likely to vary substantially by age and gender, and we did not have enough observations to run the simulation by age-gender subgroups. The findings on estimated changes in productivity by age and gender were incorporated in the Markov decision model. Additional assumptions included: workers missed an average of 40 work days to recover from THA [12]; initial postsurgery year total costs were a combination of medical costs and 50% of societal savings; and all patients retired by the age of 75 years (ie, no indirect cost benefits accrue after 74 years).

The study was approved by the institutional review board in both practices. Detailed descriptions of patient-reported outcome data, the estimated relationship between functional limitations and indirect costs, and the indirect cost assumptions are provided (Appendix 1. Supplemental material is available with the online version of *CORR* ^®^.)

#### Direct Costs

We used the Medicare Carrier, Home Health Agency, Inpatient, Outpatient, and Skilled Nursing Facility 5% sample Limited Data Sets [8] from 2009 to 2011 to estimate direct costs for THAs and revision THAs. Patients who received THA were identified using ICD-9 procedure code 81.51 with a principal diagnosis of hip osteoarthritis (ICD-9 diagnosis code 715.x5). We identified instances of revision hip replacement using ICD-9 procedure code 81.53. The sample was limited to patients with Medicare Part A and Part B coverage. We excluded patients who died during the episode period or who had multiple primary hip replacements.

Our direct cost estimate for surgical patients was computed by averaging total payments 1 year after surgery. We assumed that, if these patients had not received surgery, their healthcare utilization in the subsequent year would equal prior-year utilization. For revision, we computed direct costs for 3 months following revision, and assumed that direct costs for the remainder of the year resembled those of patients who had primary hip replacement. Direct costs include spending for services provided in hospitals (inpatient and outpatient), home, physician offices, and postacute care settings. Payments were inflated to 2011 US dollars using the Medicare standardized amounts and conversion factors of each setting. Medicare payments were converted to all-payer payment rates using a conversion factor. We assumed a successful primary or revision THA reduced direct medical costs related to symptomatic osteoarthritis by USD 590.

### Utilities

We obtained utilities from Mota [29] for five of the health states in our study: (1) nonsurgery; (2) end-stage hip OA; (3) more severe OA; (4) successful post-THA; (5) post-first revision THA.

These utilities, synthesized by Mota [29] in a literature review, were based on studies researching individual patient data of quality of life with the EuroQol five-dimensional (EQ-5D) questionnaire. We calculated the utilities for the other two health states: (1) initial post-THA and (2) post-second THA revision. The utility of initial post-THA state was a combination of 25% of the pre-THA utility and 75% of post-THA utility. The utility of post-second THA revision was calculated based on the assumption that post-second THA revision utility could reach 90% of the utility for post-first THA revision (Table 1).

**Table 1:** Transition probabilities, utilities, and medical costs in the Markov model for THA

Parameter	Value	Source
Mortality		
Natural death	Varies by age and gender	US Census Bureau Life Expectancy Table [39]
Perioperative THA death	Varies by age and gender	Memtsoudis et al. [27]
Perioperative revision death	Age < 75 years: 0.003; age ≥ 75 years: 0.012	Mota [29]
Revision		
Early aseptic first revision	Varies by age and gender	Australian Orthopaedic Association National Joint Replacement Registry [1]; Parvizi et al. [32]
Late aseptic first revision	Varies by age and gender	Australian Orthopaedic Association National Joint Replacement Registry [1]; Parvizi et al. [32]
Early aseptic second revision	0.0583	Australian Orthopaedic Association National Joint Replacement Registry [1]; Parvizi et al. [32]
Late aseptic second revision	0.022	Australian Orthopaedic Association National Joint Replacement Registry [1]; Parvizi et al. [32]
Early infection first revision	Varies by age and gender	Australian Orthopaedic Association National Joint Replacement Registry [1]; Parvizi et al. [32]
Late infection first revision	Varies by age and gender	Australian Orthopaedic Association National Joint Replacement Registry [1]; Parvizi et al. [32]
Early infection second revision	0.017	Australian Orthopaedic Association National Joint Replacement Registry [1]; Parvizi et al. [32]
Late infection second revision	0.006	Australian Orthopaedic Association National Joint Replacement Registry [1]; Lie et al. [23]
Transitional probability of nonoperative treatment to more severe OA		
More severe OA	0.041	Mota [29]
Utility		
End-stage hip OA	Varies by gender	Value for males: 0.52; for females: 0.47; Mota [29]
Nonsurgery	Varies by gender	Value for males: 0.52; for females: 0.47; Mota [29]
More severe OA	0.28	Mota [29]
Initial post-THA	0.74	Mota [29]; current authors’ calculation
Successful post-THA	Varies by gender	Values for males: 0.83; for females: 0.8; Mota [29]
Post-first revision THA (early and late)	0.64	Mota [29]
Post-second THA revision	0.58	Mota [29]; current authors’ calculation
Annual direct medical costs		
End-stage OA/nonsurgery/more severe OA	USD 12,815	Current authors’ calculation
Initial post-THA	USD 38,965	Current authors’ calculation
Successful THA	USD 12,225	Base value: medical cost of end-stage OA USD 590; current authors’ calculation
First aseptic revision THA (early and late)	USD 57,141	Current authors’ calculation
First infection revision THA (early and late)	USD 95,763	Current authors’ calculation

OA = osteoarthritis.

### Health State Transition and Transitional Probabilities

The transition probabilities for the nonsurgical treatment arm were directly extracted from existing literature or statistics. Specifically, the transition probability from end-stage hip OA to more severe OA, and the probability of death at each cycle were extracted from Mota [29] and the US life tables [39], respectively.

The perioperative revision mortality probabilities were extracted from Mota [29], based on a literature review. Mota synthesized findings of US-based THA revision studies that involved large US patient databases—US Medicare claims and National Inpatient Sample, and risk adjustment by patient characteristics. For the perioperative THA mortality, we conducted a systematic literature review to obtain mortality rates for more granular age categories than the ones presented by Mota [29]. We used the risk-adjusted mortality rates reported by Memtsoudis et al. [27], which had the most granular age categorization based on our literature review (Table 1). Revision rates were obtained from the Australian Orthopaedic Association National Joint Replacement Registry [1] (Table 1). We used the Australian joint replacement registry because it was the only registry that contained OA-specific THA revision rates by age and gender. In addition to the Australian registry, we also searched the Swedish, Norwegian, and British registries. The US registry was launched in 2012, so it does not contain the late revision rates that we need for our model.

Early revision rates were modeled as revision surgery in the year after index THA, whereas late failure was modeled as revision surgery in subsequent years. We also differentiated between a patient’s first and subsequent revisions, and aseptic and infection revisions. The first-time aseptic revision rate was 6.69 times the first-time infection revision rate [1] and the second aseptic revision rate was 3.4 times the second infection revision rate [32]. We estimated the first revision rate by age weighted on the gender distribution of all patients who had THA in 2011.

### Sensitivity Analysis

We conducted threshold analyses to identify the value of key parameters where net savings equal zero. In addition, we performed Monte Carlo analysis to evaluate: (1) the effect of uncertainty in the model assumptions regarding indirect cost benefits using probabilistic sensitivity analysis and (2) the effect of individual patient variability in age and gender using microsimulation.

## Results

### Expected Effect of THA on Patients’ Employment and Earnings

We estimated that THA increases the overall likelihood of employment by 18% (95% CI, 12%-23%). Our simulation found no difference in average annual earnings among workers who underwent THA relative to those who pursued nonsurgical management (mean change in earnings, USD 5073; 95% CI, USD −3188 to USD 13,387). Overall, we estimated that the average annual change in productivity, which reflected changes in the likelihood of employment and earnings, increased by USD 9503 for patients undergoing THA (95% CI, USD 1446–USD 17,812). We also estimated that THA reduces missed work days in a year by 36 days, from 50 to 14 missed work days (95% CI, 12–75 days). This estimate of missed work days did not incorporate missed work days to recover from THA, which we assumed was 40 days.

We estimated the effects of THA by age and gender (Table 2). The results showed changes in estimated annual earnings, value of missed work, and productivity for patients undergoing THA compared with nonoperative treatment. The expected effects of THA on productivity ranged from USD 15,830 (males 40 to 49 years old) to USD 1917 (females 70 to 74 years old). The wide variation by age and, to a lesser extent, gender largely reflected differences in labor force participation rates (and likelihood of being employed) in these categories.

**Table 2:** Annual change in earnings and value of missed work

Gender/age	Change in earnings (in USD)	Change in value of missed work (in USD)	Total change in productivity (in USD)
Male			
40–49 years	14,486	1344	15,830
50–59 years	15,408	991	16,399
60–64 years	14,021	397	14,419
65–69 years	9336	79	9415
70–74 years	4283	–9	4274
≥75 years*	0	0	0
Female			
40–49 years	11,781	761	12,542
50–59 years	12,352	559	12,911
60–64 years	9930	167	10,097
65–69 years	5477	−6	5470
70–74 years	1927	−10	1917
≥ 75 years*	0	0	0

Values represent the 1-year difference in earnings or value of missed work between THA and nonoperative treatment options; *we assumed individuals retire by 75 years and therefore surgery results in no change in earnings, missed work days, or total productivity.

### Cost-effectiveness of THA Relative to Nonsurgical Treatment

Using our Markov model, we estimated that mean lifetime direct cost for THA per patient was USD 30,365 higher than nonoperative treatment (Table 3). The incremental direct costs associated with surgery were offset by USD 63,314 in indirect cost savings for a net societal savings of USD 32,948. Therefore, THA was a dominant treatment strategy relative to nonoperative treatment because it resulted in overall cost savings with 5.5 more QALYs. Although the results showed net societal benefits from THA in the entire cohort, direct medical costs began to exceed indirect cost savings after patients were 64 years old. THA was cost-effective for all age groups at traditional willingness-to-pay thresholds, with the highest incremental cost per QALY gained at USD 10,748 for patients 75 years old or older.

**Table 3:** Summary of the lifetime costs and savings from THA by age

Age group	Incremental direct cost (A) (in USD)	Incremental indirect cost savings (B) (in USD)	Net societal savings (C) (in USD)	QALY gained (D)	ICER (C/D)
< 65 years	28,067	125,958	97,892	7.0	Dominant
40–49 years	28,905	218,738	189,833	8.3	Dominant
50–59 years	28,128	136,023	107,894	7.1	Dominant
60–64 years	27,564	65,659	38,095	6.0	Dominant
≥ 65 years	32,323	10,052	−22,271	4.2	−5255
65–69 years	30,147	28,157	−1990	5.4	−371
70–74 years	30,162	5664	−24,497	4.4	−5618
≥ 75 years	35,468	0	−35,468	3.3	−10,748
All	30,365	63,314	32,948	5.5	Dominant

ICER = incremental cost-effectiveness ratio; Net societal savings = Column B minus Column A; QALY = quality adjusted life year; weights by gender in age groups were calculated using 2011 National Inpatient Sample data.

The results showed that younger patients receiving THA accrued substantially higher lifetime savings because of the longer length of time in the workforce. The difference in indirect cost savings between the youngest group (40–49 years old) and patients between 70 and 74 years old was USD 213,074 over a lifetime (Table 3). The average net societal savings for patients younger than 65 years was USD 97,892, while no societal savings were obtained for patients 65 years and older.

### Sensitivity Analyses

We performed a threshold analysis to estimate the sensitivity of societal savings from THA to key model assumptions for patients 55 and 60 years old (Table 4). The results were robust to many of the model assumptions; we observed societal savings even after decreasing or increasing the base case assumptions (Table 4) by a factor of 10. With respect to productivity, the breakeven points for a female (male) 55 and 60 years old were 26% (20%) and 41% (28%) of base case assumptions (Table 1) (Appendix Table 3. Supplemental material is available with the online version of *CORR* ^®^).

**Table 4:** Base case and threshold values for zero net societal savings

Parameter	Base case (male, female)	Age 55 years	Age 60 years
		Threshold	Threshold
First aseptic revision rate (early)	1.31%, 1.13%	Robust	Robust
First aseptic revision rate (late)	0.61%, 0.63%	Robust	Robust
First infection revision rate (early)	0.20%, 0.17%	Robust	Robust
First infection revision rate (late)	0.09%	Robust	Robust
End-stage osteoarthritis [1]	USD 12,815	Robust	Robust
Initial post-THA (365 days)	USD 38,965	USD 123,700 (female) USD 170,100 (male)	USD 78,800 (female) USD 116,900 (male)
Productivity	Varies by age and gender	26% of base (female) 20% of base (male)	41% of base (female) 28% of base (male)

Parameters are considered robust if net savings remain at 10 times the value of the base case; direct cost of end-stage osteoarthritis includes all medical costs, not just those associated with treatment of osteoarthritis.

**Fig. 2:**
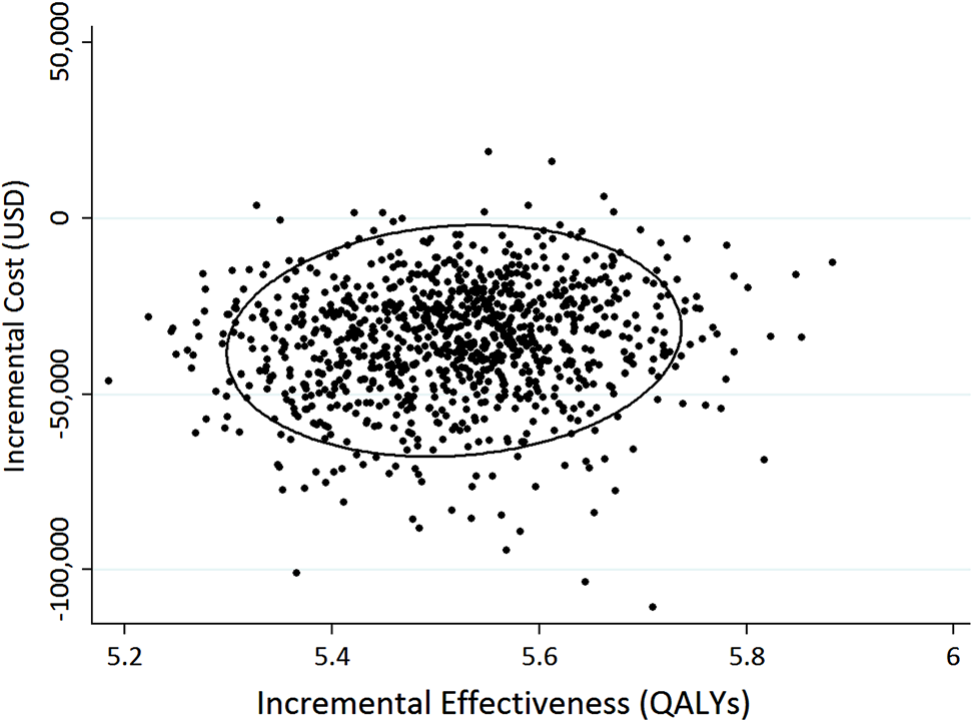
The Y-axis and X-axis values of each dot correspond to the incremental cost and incremental effectiveness (THA vs nonsurgery) for a sampling of 1000 average American patients (mean age, 66 years; sex, 42% women). The dispersion illustrates the effect of uncertainty of the indirect cost parameters, which follow logistic and lognormal distribution for male and female patients respectively. QALYs = quality adjusted life years

We used a Monte Carlo simulation to perform a probabilistic sensitivity analysis to assess the effect of parameter uncertainty in the indirect cost benefits. The mean lifetime cost for THA was USD 142,975 (95% CI, USD 141,960 –USD 143,990) and the mean cost of nonoperative treatment was USD 177,875 (95% CI, USD 177,700–USD 178,051) for a mean net benefit of USD 34,900 (95% CI, USD 33,880–USD 35,920). For a typical patient in the US healthcare system, THA was the cost-saving strategy in more than 95% of trials (Fig. 2). The ellipse in that figure captured the 95% CI for the difference between the means of each strategy.

## Discussion

Demand for THA among working-age patients has contributed to the growth in surgeries during the last decade. According to the Healthcare Cost and Utilization Project statistics based on the Nationwide Inpatient Sample [16], the number of THAs performed on patients 18 to 64 years old has increased by 91% from 100,376 to 192,105 between 2003 and 2013. The potential benefits of THA include greater work participation and earnings and fewer missed work days. The societal effect of THA depends on these indirect cost benefits from surgery. To our knowledge, our study is the first to incorporate these potential indirect cost benefits of surgery in a model on the cost-effectiveness of THA. Because patients are receiving THA at younger ages, the inclusion of employment and earnings benefits results in a more accurate assessment of the true societal effects of surgery. Our study shows that after accounting for productivity gains from THA, THA is superior to nonsurgical procedures for patients with hip OA in terms of societal value. On average, THA results in net societal savings of USD 32,948 per patient and adds 5.5 QALYs over a patient’s lifespan.

The current study has several limitations. First, we inferred the effects of THA on employment, earnings, and missed work days by using the estimated relationship between functional limitations and economic outcomes for a sample of NHIS respondents and linking these estimates to patient-reported outcome for a sample that underwent THA. Although this technique followed prior research [11, 35], the method is based on the assumption that treatments only affect productivity through their impact on functionality. This approach takes the perspective of an average working patient and, thus, may not be applicable to patients in a specific occupation.

Second, to estimate the post-THA productivity, the patient-reported outcome functionality data were collected retrospectively from patients up to 2 years after THA. Our sample was small and response rates were low (30%), which raises concerns regarding the generalizability of the findings. In addition, our retrospective approach introduces the possibility of recall bias regarding functional limitations before surgery. To assess potential recall bias, we compared baseline functional status values with published values [3, 14, 17, 31, 33]. Using a functional limitations index scale (ranging from 0 to 100, with higher values indicating greater function impairment), we found an average index score of 54 before THA based on our collected patient-reported outcome data. Our sample had similar preoperative function compared with those observed in previous studies, which ranged from 55 to 63 (of 100) in prospective studies using the WOMAC functional score [3, 14, 17, 31, 33]. Third, we assumed that the productivity of the nonsurgical group would be maintained at the presurgery values. Fourth, we used summary statistics from the Australian Joint Replacement Registry [1] to estimate the early and late revision rates. The revision rates from the Australian Orthopaedic Association National Joint Registry may serve as only a limited proxy for the US revision rates to the extent that patient demographic and clinical characteristics and joint replacement technologies differ between the two countries. Fifth, the economic and societal benefits of THA might be undervalued because we do not incorporate the value of nonpaid work, such as child care. Finally, our study focuses on the societal values of typical THAs performed in the inpatient setting.

In this study, we explored the effects of THA on employment, earnings, and missed work days and then assessed how these effects affect the measured cost-effectiveness of THA. We estimated increases in the likelihood of being employed (from 39% to 57%) and annual productivity (USD 9503). The study findings showed the importance of work-related benefits from THA. Conceptually, the potential effects of continued end-stage OA of the hip versus THA on workplace productivity seem clear. People with hip pain and associated functional limitations may be unable to work or may need to work a part-time or more limited schedule. Among people with end-stage OA who are able to maintain full-time employment, the effects on productivity have the potential to negatively influence their earnings. Empirical evidence on the effect of end-stage OA and THA on employment is limited, but generally supportive of the view that THA allows patients to maintain employment [4]. Numerous studies have shown that the majority of patients having THA return to work after the surgery [5, 10, 13, 30, 38]. For those returning to work, most do not encounter functional limitations in their ability to fully engage in work activities [30, 36]. Two studies found that some patients who are out of work before surgery regained employment after THA [4, 28].

After incorporating the effects of THA on productivity, we showed a positive societal rate of return on healthcare spending for THA. We estimated that THA results in incremental direct costs of USD 30,365, which were offset by USD 63,314 in lifetime savings from increased productivity, for an average net societal savings of USD 32,948. We found that the estimated lifetime societal net benefit is comparable to that estimated for TKA (USD 18,930; USD 39,565 indirect cost benefits and USD 20,635 direct medical costs) [35].

Societal savings from THA were greater for younger patients because of their higher remaining years in the workforce, with a breakeven point at age 65 years. Findings on overall cost savings of THA were robust to model assumptions for patients younger than 60 years. For patients 65 years or older, the existence of societal savings was sensitive to revision rates, medical costs of primary and aseptic revision THAs, and indirect cost savings. Nevertheless, the procedure is highly cost-effective regardless of age when using standard thresholds of incremental cost per QALY, findings consistent with those of prior studies [9, 29]. We estimated a total life societal savings of almost USD 10 billion from the more than 300,000 THAs performed in the United States each year. Our study concludes that THA produces net societal savings because of its effect on younger patients’ work status and earnings. Policy makers and payers should consider the indirect cost benefits of THA when assessing policies to control healthcare spending and improve quality. Given the positive return to society from spending on THA, as shown in our model results, policy proposals that discourage decisions to choose THA may be counterproductive.

## Electronic supplementary material

Below is the link to the electronic supplementary material.

Supplementary material 1 (DOC 264 kb)

## References

[1] Australian Orthopaedic Association National Joint Replacement Registry. Hip and Knee Arthroplasty. Annual Report 2013. Available at: https://aoanjrr.sahmri.com/documents/10180/127202/Annual%20Report%202013?version=1.2&t=1385685288617. Accessed June 19, 2014.

[2] Babovic N, Trousdale RT (2013). Total hip arthroplasty using highly cross-linked polyethylene in patients younger than 50 years with minimum 10-year follow-up. J Arthroplasty..

[3] Bachmeier CJ, March LM, Cross MJ, Lapsley HM, Tribe KL, Courtenay BG (2001). Brooks PM; Arthritis Cost and Outcome Project Group. A comparison of outcomes in osteoarthritis patients undergoing total hip and knee replacement surgery. Osteoarthritis Cartilage..

[4] Bieleman HJ, Bierma-Zeinstra SM, Oosterveld FG, Reneman MF, Verhagen AP, Groothoff JW (2011). The effect of osteoarthritis of the hip or knee on work participation. J Rheumatol..

[5] Bohm ER (2010). The effect of total hip arthroplasty on employment. J Arthroplasty..

[6] Caton J, Prudhon JL (2011). Over 25 years survival after Charnley’s total hip arthroplasty. Int Orthop..

[7] CDC Centers for Disease Control and Prevention. National Health Interview Survey. Available at: https://www.cdc.gov/nchs/nhis/quest_data_related_1997_forward.htm. Accessed June 19, 2014.

[8] Centers for Medicare & Medicaid Services. Limited Data Set Files. Available at: https://www.cms.gov/Research-Statistics-Data-and-Systems/Files-for-Order/LimitedDataSets/index.html. Accessed June 19, 2014.

[9] Chang RW, Pellisier JM, Hazen GB (1996). A cost-effectiveness analysis of total hip arthroplasty for osteoarthritis of the hip. JAMA..

[10] Cowie JG, Turnbull GS, Turnball GS, Ker AM, Breusch SJ (2013). Return to work and sports after total hip replacement. Arch Orthop Trauma Surg..

[11] Dall TM, Gallo P, Koenig L, Gu Q, Ruiz D (2013). Modeling the indirect economic implications of musculoskeletal disorders and treatment. Cost Eff Resour Alloc..

[12] Denniston PL, Kennedy CW (2011). 2012 Official Disability Guidelines.

[13] Glebus GP, Feather TW, Hsu JR, Gerlinger TL (2013). Return to duty and deployment after major joint arthroplasty. J Arthroplasty..

[14] Hamel MB, Toth M, Legedza A, Rosen MP (2008). Joint replacement surgery in elderly patients with severe osteoarthritis of the hip or knee: decision making, postoperative recovery, and clinical outcomes. Arch Intern Med..

[15] HCUP Agency for Healthcare Research and Quality. Overview of the National (Nationwide) Inpatient Sample (NIS). Available at: https://www.hcup-us.ahrq.gov/nisoverview.jsp. Accessed June 19, 2014.

[16] HCUP Agency for Healthcare Research and Quality. HCUPnet. Rockville, MD: Agency for Healthcare Research and Quality. Date last modified December 11, 2015. Available at: https://hcupnet.ahrq.net.gov/. Accessed June 19, 2014.

[17] Jones CA, Voaklander DC, Johnston DW, Suarez-Almazor ME (2001). The effect of age on pain, function, and quality of life after total hip and knee arthroplasty. Arch Intern Med..

[18] Koenig L, Dall TM, Gu Q, Saavoss J, Schafer MF (2014). How does accounting for worker productivity affect the measured cost-effectiveness of lumbar discectomy?. Clin Orthop Relat Res..

[19] Kurtz SM, Lau E, Ong K, Zhao K, Kelly M, Bozic KJ (2009). Future young patient demand for primary and revision joint replacement: national projections from 2010 to 2030. Clin Orthop Relat Res..

[20] Kurtz SM, Ong KL, Lau E, Bozic KJ (2014). Impact of the economic downturn on total joint replacement demand in the United States: updated projections to 2021. J Bone Joint Surg Am..

[21] Laupacis A, Bourne R, Rorabeck C, Feeny D, Wong C, Tugwell P, Leslie K, Bullas R (1994). Costs of elective total hip arthroplasty during the first year: cemented versus noncemented. J Arthroplasty..

[22] Lavernia CJ, Alcerro JC (2011). Quality of life and cost-effectiveness 1 year after total hip arthroplasty. J Arthroplasty..

[23] Lie SA, Havelin LI, Furnes ON, Engesaeter LB, Vollset SE (2004). Failure rates for 4762 revision total hip arthroplasties in the Norwegian Arthroplasty Register. J Bone Joint Surg Br..

[24] Mariconda M, Galasso O, Costa GG, Recano P, Cerbasi S (2011). Quality of life and functionality after total hip arthroplasty: a long-term follow-up study. BMC Musculoskelet Disord..

[25] Mather RC, Koenig L, Acevedo D, Dall TM, Gallo P, Romeo A, Tongue J, Williams G (2013). The societal and economic value of rotator cuff repair. J Bone Joint Surg Am..

[26] Mather RC, Koenig L, Kocher MS, Dall TM, Gallo P, Scott DJ, Bach BP, Spindler KP (2013). Societal and economic impact of anterior cruciate ligament tears. J Bone Joint Surg Am..

[27] Memtsoudis SG, Pumberger M, Ma Y, Chiu YL, Fritsch G, Gerner P, Poultsides L, Valle AG (2012). Epidemiology and risk factors for perioperative mortality after total hip and knee arthroplasty. J Orthop Res..

[28] Mobasheri R, Gidwani S, Rosson JW (2006). The effect of total hip replacement on the employment status of patients under the age of 60 years. Ann R Coll Surg Engl..

[29] Mota RE (2013). Cost-effectiveness analysis of early versus late total hip replacement in Italy. Value Health..

[30] Nunley RM, Ruh EL, Zhang Q, Della Valle CJ, Engh CA, Berend ME, Parvizi J, Clohisy JC, Barrack RL (2011). Do patients return to work after hip arthroplasty surgery. J Arthroplasty..

[31] Ostendorf M, van Stel HF, Buskens E, Schrijvers AJ, Marting LN, Verbout AJ, Dhert WJ (2004). Patient-reported outcome in total hip replacement: a comparison of five instruments of health status. J Bone Joint Surg Br..

[32] Parvizi J, Pour AE, Keshavarzi NR, D’Apuzzo M, Sharkey PF, Hozack WJ (2007). Revision total hip arthroplasty in octogenarians: a case-control study. J Bone Joint Surg Am..

[33] Patil S, Garbuz DS, Greidanus NV, Masri BA, Duncan CP (2008). Quality of life outcomes in revision vs primary total hip arthroplasty: a prospective cohort study. J Arthroplasty..

[34] Räsänen P, Paavolainen P, Sintonen H, Koivisto AM, Blom M, Ryynänen OP, Roine RP (2007). Effectiveness of hip or knee replacement surgery in terms of quality-adjusted life years and costs. Acta Orthop..

[35] Ruiz D, Koenig L, Dall TM, Gallo P, Narzikul A, Parvizi J, Tongue J (2013). The direct and indirect costs to society of treatment for end-stage knee osteoarthritis. J Bone Joint Surg Am..

[36] Sankar A, Davis AM, Palaganas MP, Beaton DE, Badley EM, Gignac MA (2013). Return to work and workplace activity limitations following total hip or knee replacement. Osteoarthitis Cartilage..

[37] Schenker N, Raghunathan TE, Chiu P, Makuc DM, Zhang G, Cohen AJ (2006). Multiple imputation of missing income data in the National Health Interview Survey. J Am Stat Assoc..

[38] Tilbury C, Schaasberg W, Plevier JW, Fiocco M, Nelissen RG (2014). Vliet Vlieland TP. Return to work after total hip and knee arthroplasty: a systematic review. Rheumatology..

[39] United States Census Bureau. Statistical Abstract of the United States 2012: Table 104 Expectation of Life at Birth, 1960 to 2008, and Projections, 2010 to 2020. Available at: https://www.census.gov/library/publications/2011/compendia/statab/131ed/tables/12s0104.xls. Accessed June 19, 2014.

