# Supplementary material for: Estimating the Societal Benefits of THA After Accounting for Work Status and Productivity: A Markov Model Approach
Source: Clin Orthop Relat Res. 2016 Oct 3;474(12):2645–54. doi: 10.1007/s11999-016-5084-9 (PMC5085951; doi:10.1007/s11999-016-5084-9)
Supplement: Supplementary file 1 — Supplementary material 1 (DOC 264 kb) [file 11999_2016_5084_MOESM1_ESM.doc]

**Appendix 1.** Supplementary data and results

Contents

1. Converting Medicare Costs to All-payer Costs
2. Changes in Direct Costs After THA
3. Patient-reported Outcomes Data
4. Estimating the Relationship Between Functional Limitations and Indirect Costs
5. Revision Rates and Perioperative Mortality Rates by Age and Gender
6. Cost-effectiveness of THA Without Indirect Costs by Age
7. Cost-effectiveness of THA by Age and Gender
8. Acronyms and Definitions
9. Converting Medicare Costs to All-payer Costs

Cost estimates based on Medicare payment rates may underestimate payments made by private insurers and overestimate payments made by Medicaid, self-insured, and uninsured patients. To reconcile these differences, we adjusted our estimates of direct medical costs using payment rates of other insurers (as a percentage of the Medicare rate) and then weighted by the national distribution of payers for treatment of THA. We set the payment rate of Medicaid and self-pay patients as 80% and 50% of the Medicare rate, respectively. For private insurers, we used the payment rates reported in the literature. White et al. [3] estimated that private insurers, on average, paid 150% of the Medicare payment rates for inpatient care nationally in 2011. They also reported private insurer payments were two times the Medicare rate for outpatient services. The Medicare Payment Advisory Committee estimated that the private rate for physician services was, on average, 123% of the Medicare rate across all services and areas in 2003 [2]. For all other patients, including those paid by workers’ compensation, we assumed their rate was the same as the average rates of Medicare and private insurers.

1. Changes in Direct Costs After THA

We assumed that patients with a successful THA would have two fewer physician visits per year (2*USD 112), an annual reduction in osteoarthritis (OA)-related medical equipment (including canes, walkers, crutches, footwear, splints, braces, and wheelchairs) (USD 7.05) [1], and 75% lower annual costs for OA medications owing to pain management (USD 358.82). As a result, health care costs for patients with a successful THA would be reduced by USD 590 per year.

Based on our analysis, the unit cost for a physician visit was estimated to be USD 112 per visit in 2011. The annual OA-related medical equipment was USD 6.50 in 2007, and total annual costs for OA medications were USD 441 in 2007 [3]. The two values then were adjusted to 2011 US dollars, USD 7.05 for OA-related medical equipment costs and USD 358.82 for OA medication costs.

1. Patient-reported Outcomes Data

Predicted values for indirect cost components were obtained by using patient-reported data collected by two physician group practices with multiple locations in the northeast. Electronic questionnaires were sent to 260 patients who underwent THA between September 2010 and April 2014. A total of 90 responses were received. The analysis included 77 complete responses for patients undergoing the first THA for OA.

The survey contained questions concerning an individual’s socioeconomic status and functional ability (using the same questions on functional status in the National Health Interview Survey (NHIS) before undergoing surgery and after having surgery. Functional questions included the following possible answers: “No Difficulty”, “Only A Little Difficult”, “Somewhat Difficult”, “Very Difficult”, and “Cannot Do.” Numerical values of 0 through 4 were assigned to the responses, with “No difficulty” assigned a value of 0 and “Cannot do” assigned a value of 4. An index of functional status was established by adding the functional scores for each item and converting to a 100 scale. The data contain 55% males and 45% females. The patients were 60 years old on average with a range of 29 to 80 years. Mean response scores are shown in Table 1, and descriptive statistics of the patient demographics are shown in Table 2.

**Table 1.** Mean change in functional status of THA from patient responses (n = 77)*

| Questions | Presurgery responses | Postsurgery responses |
| --- | --- | --- |
| Walk a quarter of a mile | 2.65 | 0.43 |
| Walk up 10 steps | 2.23 | 0.23 |
| Sit for about 2 hours | 2.08 | 0.45 |
| Stand or be on your feet for about 2 hours | 2.64 | 0.75 |
| Stoop, bend, or kneel | 2.70 | 0.91 |
| Lift or carry 10 pounds | 2.06 | 0.43 |
| Push or pull large objects (eg, chair) | 2.19 | 0.68 |

*All differences between mean presurgery and postsurgery scores were significant at the p < 0.001 level.

**Table 2.** Patient demographic and functionality characteristics

| Variable | NHIS | Preoperative patient-reported outcome | Postoperative patient-reported outcome |
| --- | --- | --- | --- |
| Sample size | 34,112 | 77 | 77 |
| Age |  |  |  |
| 16-19 years | 2.1% | 0.0% | 0.0% |
| 20-29 years | 16.5% | 1.3% | 1.3% |
| 30-39 years | 17.4% | 3.9% | 3.9% |
| 40-49 years | 16.4% | 7.8% | 7.8% |
| 50-59 years | 17.5% | 33.8% | 33.8% |
| 60-69 years | 15.1% | 32.5% | 32.5% |
| 70-79 years | 9.3% | 19.5% | 19.5% |
| 80-89 years | 5.8% | 1.3% | 1.3% |
| Gender |  |  |  |
| Female | 55.3% | 45.5% | 45.5% |
| Male | 44.7% | 54.5% | 54.5% |
| Functional status | | | |
| Difficulty carrying objects |  | | |
| No difficulty | 86.0% | 11.7% | 67.5% |
| Only a little difficult | 3.9% | 20.8% | 23.4% |
| Somewhat difficult | 3.4% | 24.7% | 7.8% |
| Very difficult | 2.3% | 35.1% | 1.3% |
| Cannot do at all | 4.4% | 7.8% | 0.0% |
| Difficulty climbing stairs |  | | |
| No difficulty | 83.7% | 5.2% | 83.1% |
| Only a little difficult | 4.7% | 13.0% | 10.4% |
| Somewhat difficult | 3.9% | 40.3% | 6.5% |
| Very difficult | 2.9% | 36.4% | 0.0% |
| Cannot do at all | 4.8% | 5.2% | 0.0% |
| Difficulty pushing/pulling large objects |  | | |
| No difficulty | 80.9% | 13.0% | 54.5% |
| Only a little difficult | 4.5% | 14.3% | 26.0% |
| Somewhat difficult | 4.1% | 26.0% | 16.9% |
| Very difficult | 2.7% | 33.8% | 2.6% |
| Cannot do at all | 7.9% | 13.0% | 0.0% |
| Difficulty reaching |  | | |
| No difficulty | 89.2% | 58.4% | 93.5% |
| Only a little difficult | 4.0% | 22.1% | 3.9% |
| Somewhat difficult | 3.4% | 9.1% | 1.3% |
| Very difficult | 1.9% | 10.4% | 1.3% |
| Cannot do at all | 1.6% | 0.0% | 0.0% |
| Difficulty sitting |  | | |
| No difficulty | 86.8% | 7.8% | 67.5% |
| Only a little difficult | 4.7% | 19.5% | 19.5% |
| Somewhat difficult | 4.0% | 35.1% | 13.0% |
| Very difficult | 2.2% | 32.5% | 0.0% |
| Cannot do at all | 2.3% | 5.2% | 0.0% |
| Difficulty standing |  | | |
| No difficulty | 76.1% | 5.2% | 41.6% |
| Only a little difficult | 5.7% | 5.2% | 44.2% |
| Somewhat difficult | 5.1% | 27.3% | 11.7% |
| Very difficult | 4.4% | 45.5% | 2.6% |
| Cannot do at all | 8.7% | 16.9% | 0.0% |
| Difficulty kneeling |  |  |  |
| No difficulty | 72.4% | 0.0% | 41.6% |
| Only a little difficult | 8.5% | 10.4% | 32.5% |
| Somewhat difficult | 7.3% | 23.4% | 19.5% |
| Very difficult | 5.9% | 53.2% | 6.5% |
| Cannot do at all | 5.9% | 13.0% | 0.0% |
| Difficulty walking |  |  |  |
| No difficulty | 78.4% | 3.9% | 72.7% |
| Only a little difficult | 5.4% | 6.5% | 18.2% |
| Somewhat difficult | 4.9% | 26.0% | 3.9% |
| Very difficult | 3.7% | 48.1% | 3.9% |
| Cannot do at all | 7.5% | 15.6% | 1.3% |
| Limitations in kind or amount of work |  |  |  |
| Not limited | 85.1% | 53.2% | 89.6% |
| Moderately limited | 4.9% | 33.8% | 7.8% |
| Unable to work | 10.0% | 13.0% | 2.6% |

D. Estimating the Relationship Between Functional Limitations and Indirect Costs

The NHIS collects information from a stratified random sample of the US population on physical function, economic factors such as employment status and income, and other patient characteristics. Our analysis used data from the 2013 NHIS, with a sample of 34,112.

The relationship between earnings and functional status was estimated using a two-stage Heckman selection model to correct for potential bias created because earnings are not observed for nonemployed individuals. We used a probit model to predict employment and a linear regression equation to predict earnings. The missed work days model was estimated using a negative binomial equation. The coefficients from these models are shown in the table below.

**Table 3.** Regression coefficients from the National Health Interview Survey

| Parameter | Employment | Earnings (USD) | Missed work days |
| --- | --- | --- | --- |
|
|  |
| Intercept | 0.3368*** | -1880 | 0.4464*** |
| Gender | | | |
| Male | 0.2092*** | 12,233*** | -0.1106*** |
| Age (years) | | | |
| 20-29 vs 16-19 | 0.6193*** | 10,578*** | 0.3635*** |
| 30-39 vs 16-19 | 0.7199*** | 22,429*** | 0.5224*** |
| 40-49 vs 16-19 | 0.7722*** | 27,715*** | 0.5647*** |
| 50-59 vs 16-19 | 0.6299*** | 29,679*** | 0.3529*** |
| 60-64 vs 16-19 | 0.1511** | 24,420*** | 0.2676* |
| 65-69 vs 16-19 | -0.4902*** | 17,936*** | 0.5541*** |
| 70-79 vs 16-19 | -1.0842*** | 9570*** | -0.4055** |
| 80-89 vs 16-19 | -1.6624*** | 2391 | -1.5278*** |
| Ethnicity | | | |
| Asian vs white | -0.4134*** | -2894*** | -0.2611*** |
| Black vs white | -0.1560*** | -4744*** | -0.0121 |
| Hispanic vs white | -0.1139*** | -6021*** | -0.1022** |
| Other vs white | -0.1287** | -3604** | 0.3056** |
| Marital status | | | |
| Never married vs married | -0.2507*** | -5467*** | 0.0842** |
| Widowed/divorced/separated  vs married | -0.1478*** | -1046** | 0.1102*** |
| Family size | | | |
| 1 child vs no children | 0.0537* | 961* | 0.0160 |
| 2 children vs no children | -0.1006*** | 3198*** | -0.0550 |
| 3+ children vs no children | -0.3041*** | -366 | -0.1796*** |
| Education | | | |
| High school vs less than high school | 0.4172*** | 9023*** | 0.0290 |
| Associates degree vs less than high school | 0.2082*** | 5555*** | 0.0621 |
| Baccalaureate degree vs less than high school | 0.1971*** | 12,281*** | -0.1925*** |
| Postgraduate degree vs less than high school | 0.1454*** | 14,036*** | 0.0465 |
| Difficulty carrying vs no difficulty | | | |
| Only a little difficult | -0.1165** | 768 | -0.0342 |
| Somewhat difficult | -0.1724*** | 1108 | -0.0948 |
| Very difficult | -0.1156 | 5466** | 0.1642 |
| Cannot do | -0.3216*** | -2140 | 0.3774 |
| Difficulty climbing vs no difficulty | | | |
| Only a little difficult | -0.0680 | -797 | 0.069 |
| Somewhat difficult | -0.0582 | -1525 | -0.1456 |
| Very difficult | -0.0549 | -2608 | -0.2128 |
| Cannot do | -0.0595 | 82 | -0.0208 |
| Difficulty sitting vs no difficulty | | | |
| Only a little difficult | -0.1270*** | -1736 | -0.1866* |
| Somewhat difficult | -0.0966** | -2009 | 0.0592 |
| Very difficult | -0.2239*** | -3032 | -0.3215* |
| Cannot do | -0.1868*** | -1531 | -0.2587 |
| Difficulty standing vs no difficulty | | | |
| Only a little difficult | -0.2762*** | -347 | 0.5046*** |
| Somewhat difficult | -0.3031*** | -573 | 0.3252*** |
| Very difficult | -0.4448*** | -352 | 0.8851*** |
| Cannot do | -0.6114*** | -2403 | 0.6863*** |
| Difficulty kneeling vs no difficulty | | | |
| Only a little difficult | 0.0436 | -1850** | 0.2238*** |
| Somewhat difficult | 0.0848** | -2059** | 0.6662*** |
| Very difficult | 0.0945* | -1286 | 0.6893*** |
| Cannot do | 0.1574*** | -411 | 0.9574*** |
| Difficulty walking vs no difficulty | | | |
| Only a little difficult | -0.0569 | -1776* | 0.2726*** |
| Somewhat difficult | -0.1901*** | -3196** | 0.3760*** |
| Very difficult | -0.2831*** | -2766 | 0.3133* |
| Cannot do | -0.3951*** | -2138 | 0.3481** |
| Difficulty pushing vs no difficulty | | | |
| Only a little difficult | -0.0992** | -1231 | 0.6127*** |
| Somewhat difficult | -0.0744 | -2737* | 0.7067*** |
| Very difficult | -0.2471*** | -2675 | 0.7384*** |
| Cannot do | -0.2690*** | -2228 | 0.7444*** |
| Difficulty reaching vs no difficulty | | | |
| Only a little difficult | -0.0274 | -2650** | 0.6116*** |
| Somewhat difficult | -0.0598 | -2788* | 0.4569*** |
| Very difficult | -0.2037*** | -5897** | 0.2925 |
| Cannot do | -0.1109 | -8490** | 0.7929** |
| Other health conditions | | | |
| Breathing problem | -0.2172*** | 346 | 0.2585 |
| Depression | -0.3060*** | -4913*** | 0.2309 |
| Diabetes | -0.1806** | -3102 | 0.1175 |
| Heart problem | -0.2918*** | -928 | 0.1809 |
| Memory problem | -0.7681*** | -11,108*** | 0.9912*** |
| Senility problem | -0.3230 | -8411 | 0.8609 |
| Instruments | | | |
| Other family income (in USD 1000s) | -0.0000*** | N/A | N/A |
| Other sources of income | -0.2577*** | N/A | N/A |

Our analysis of the 2013 NHIS data; ***statistically significant at the 0.01 level; **significant at 0.05 level; *significant at 0.10 level; N/A = not applicable.

1. Revision Rates and Perioperative Mortality Rates by Age and Sex

**Table 4.** Revision rates and perioperative mortality rates for THA

|  | Base | |
| --- | --- | --- |
| Parameter | Males | Females |
| First aseptic revision rate (early) | | |
| < 55 | 1.04% | 1.48% |
| 55-64 | 1.31% | 1.13% |
| 65-74 | 1.22% | 1.301% |
| ≥ 75 | 1.57% | 1.31% |
| First aseptic revision rate (late) | | |
| < 55 | 0.751% | 0.917% |
| 55-64 | 0.614% | 0.631% |
| 65-74 | 0.496% | 0.480% |
| ≥ 75 | 0.446% | 0.281% |
| First infection revision rate (early) | | |
| < 55 | 0.156% | 0.220% |
| 55-64 | 0.200% | 0.170% |
| 65-74 | 0.180% | 0.200% |
| ≥ 75 | 0.230% | 0.200% |
| First infection revision rate (late) | | |
| < 55 | 0.112% | 0.140% |
| 55-64 | 0.092% | 0.090% |
| 65-74 | 0.074% | 0.070% |
| ≥ 75 | 0.067% | 0.040% |
| Perioperative mortality | | |
| < 45 | 0.027% | 0.032% |
| 45-64 | 0.031% | 0.038% |
| 65-74 | 0.061% | 0.075% |
| ≥ 75 | 0.179% | 0.218% |

F. Cost-effectiveness of THA without Indirect Costs by Age

**Table 5.** Direct medical costs and QALYs by age, sex, and treatments*

|  | Total direct costs (USD) | | | Quality adjusted life years | | |
| --- | --- | --- | --- | --- | --- | --- |
| Age (years) | No Surgery | Surgery | Difference | No Surgery | Surgery | Difference |
|  | | | | | | |
| Male  < 65 | 231,989 | 260,228 | 28,239 | 7.8 | 14.8 | 7.0 |
| 40-49 | 274,963 | 303,431 | 28,469 | 9.1 | 17.4 | 8.3 |
| 50-59 | 234,763 | 263,029 | 28,266 | 7.9 | 15.0 | 7.1 |
| 60-64 | 199,815 | 227,861 | 28,046 | 6.9 | 12.8 | 5.9 |
| ≥ 65 | 137,370 | 170,074 | 32,704 | 4.9 | 9.2 | 4.3 |
| 65-69 | 172,744 | 203,450 | 30,706 | 6.1 | 11.3 | 5.3 |
| 70-74 | 143,420 | 174,310 | 30,890 | 5.2 | 9.5 | 4.3 |
| ≥ 75 | 100,522 | 136,524 | 36,002 | 3.7 | 7.0 | 3.3 |
| All | 187,403 | 217,746 | 30,343 | 6.5 | 12.2 | 5.7 |
| Female | | | | | | |
| < 65 | 226,183 | 254,073 | 27,890 | 7.1 | 14.0 | 6.9 |
| 40-49 | 275,106 | 304,636 | 29,530 | 8.4 | 16.7 | 8.3 |
| 50-59 | 232,794 | 260,776 | 27,982 | 7.3 | 14.4 | 7.1 |
| 60-64 | 199,644 | 226,813 | 27,169 | 6.4 | 12.4 | 6.0 |
| ≥ 65 | 131,571 | 163,658 | 32,087 | 4.4 | 8.6 | 4.2 |
| 65-69 | 172,590 | 202,324 | 29,734 | 5.6 | 10.9 | 5.4 |
| 70-74 | 143,219 | 172,902 | 29,683 | 4.7 | 9.2 | 4.4 |
| ≥ 75 | 97,507 | 132,697 | 35,190 | 3.3 | 6.6 | 3.3 |
| All | 169,980 | 200,363 | 30,383 | 5.5 | 10.8 | 5.3 |

*****Results were weighted based on the incidence of THAs performed in the US in 2011.

G. Cost-effectiveness of THA by Age and Sex

**Table 6**. Summary of lifetime costs and savings from THA by age and sex*

| Age (years) | Incremental medical direct cost (A) (in USD) | Incremental societal savings (B) (in USD) | Net societal savings (C) (in USD) | Difference in QALYs (D) | ICER (C/D) |
| --- | --- | --- | --- | --- | --- |
| Male | | | | | |
| < 65 | 28,239 | 153,790 | 125,551 | 7.0 | Dominant |
| 40-49 | 28,469 | 243,926 | 215,457 | 8.3 | Dominant |
| 50-59 | 28,266 | 161,050 | 132,784 | 7.1 | Dominant |
| 60-64 | 28,046 | 83,847 | 55,801 | 5.9 | Dominant |
| > 65 | 32,704 | 15,403 | (17,301) | 4.3 | (USD 4052) |
| 65-69 | 30,706 | 38,836 | 8130 | 5.3 | Dominant |
| 70-74 | 30,890 | 8515 | (22,375) | 4.3 | (USD 5159) |
| ≥ 75 | 36,002 | 0 | (36,002) | 3.3 | (USD 10,834) |
| All | 30,343 | 88,580 | 58,237 | 5.7 | Dominant |
| Female | | | | | |
| < 65 | 27,890 | 97,473 | 69,583 | 6.9 | Dominant |
| 40-49 | 29,530 | 182,672 | 153,142 | 8.3 | Dominant |
| 50-59 | 27,982 | 109,362 | 81,380 | 7.1 | Dominant |
| 60-64 | 27,169 | 50,759 | 23,590 | 6.0 | Dominant |
| ≥ 65 | 32,087 | 6741 | (25,346) | 4.2 | (USD 6018) |
| 65-69 | 29,734 | 20,273 | (9461) | 5.4 | (USD 1767) |
| 70-74 | 29,683 | 3790 | (25,893) | 4.4 | (USD 5835) |
| ≥ 75 | 35,190 | 0 | (35,190) | 3.3 | (USD 10,573) |
| All | 30,383 | 43,574 | 13,191 | 5.3 | Dominant |

ICER = incremental cost-effectiveness ratio; *results were weighted based on the incidence of THAs performed in the US in 2011; (A), (B), (C), and (D) refer to values in corresponding columns.

H. Acronyms and Definitions

QALY = quality-adjusted life year

PRO = patient-reported outcomes

NHIS = National Health Interview Survey

NIS = National Inpatient Sample

**Definitions**

Direct costs = Direct cost refers to the medical cost incurred in the healthcare system, which is composed of primary payer payment and patient coinsurance.

Lifetime direct cost = The direct medical costs accumulated over the patient's life span for a typical patient with end-stage hip osteoarthritis since the time of confirmed diagnosis.

Productivity = In this study, productivity refers to the value of time spent in employment. We estimate productivity as expected earnings minus expected value of missed work. Expected earning is equal to the probability of working multiplied by the earnings for an individual who is employed. The value of missed work days is equal to the probability of working multiplied by the number of missed work days and an estimate of daily earnings for an individual who is working.

Societal cost = Societal cost refers to the productivity loss attributable to missed work owing to THA and postsurgical recovery.

Societal savings = Societal savings refer to the net difference in total lifetime costs between THA treatment and nonsurgical treatment. A negative difference indicates societal savings.

**References**

# 1. Berger A, Bozic K, Stacey B, Edelsberg J, Sadosky A, Oster G. Patterns of pharmacotherapy and health care utilization and costs prior to total hip or total knee replacement in patients with osteoarthritis. *Arthritis Rheum.* 2011;63:2268–2275.

2. MedPAC. Medicare Payment Advisory Commission. Report to the Congress: Medicare Payment Policy. Available at: <http://www.medpac.gov/documents/reports/Mar05_EntireReport.pdf?sfvrsn=0>. Accessed June 19, 2014.

3. White C, Bond AM, Reschovsky JD. High and varying prices for privately insured patients underscore hospital market power. *Res Brief.* 2013;27:1–10.
